# Activation of oxytocin receptors in mouse GABAergic amacrine cells modulates retinal dopaminergic signaling

**DOI:** 10.1186/s12915-022-01405-0

**Published:** 2022-09-21

**Authors:** Songhui Hu, Yurong Wang, Xu Han, Min Dai, Yongxing Zhang, Yuanyuan Ma, Shijun Weng, Lei Xiao

**Affiliations:** 1 The State Key Laboratory of Medical Neurobiology, MOE Frontiers Center for Brain Science, and the Institutes of Brain Science, Fudan University, Shanghai, 200032 China; 2 School of Biomedical Engineering, Shanghai Jiao Tong University, Shanghai, 200240 China

**Keywords:** Oxytocin receptor, Visual information processing, Retinal amacrine cells, Dopamine, Electroretinographic recording

## Abstract

**Background:**

Oxytocin, secreted by oxytocin neurons in the hypothalamus, is an endogenous neuropeptide involved in modulating multiple sensory information processing pathways, and its roles in the brain have been associated with prosocial, maternal, and feeding-related behaviors. Visual information is necessary for initiating these behaviors, with the retina consisting of the first stage in the visual system mediating external stimulus perception. Oxytocin has been detected in the mammalian retina; however, the expression and possible function of oxytocin receptors (OxtR) in the retina remain unknown. Here, we explore the role of oxytocin in regulating visual information processing in the retina.

**Results:**

We observed that OxtR mRNA and protein are expressed in the mouse retina. With Oxtr-Cre transgenic mice, immunostaining, and fluorescence in situ hybridization, we found that OxtRs are mainly expressed in GABAergic amacrine cells (ACs) in both the inner nuclear layer (INL) and ganglion cell layer (GCL). Further immunoreactivity studies showed that GABAergic OxtR^+^ neurons are mainly cholinergic and dopaminergic neurons in the INL and are cholinergic and corticotrophin-releasing hormone neurons in the GCL. Surprisingly, a high level of *Oxtr* mRNAs was detected in retinal dopaminergic neurons, and exogenous oxytocin application activated dopaminergic neurons to elevate the retinal dopamine level. Relying on in vivo electroretinographic recording, we found that activating retinal OxtRs reduced the activity of bipolar cells via OxtRs and dopamine receptors.

**Conclusions:**

These data indicate the functional expression of OxtRs in retinal GABAergic ACs, especially dopaminergic ACs, and expand the interactions between oxytocinergic and dopaminergic systems. This study suggests that visual perception, from the first stage of information processing in the retina, is modulated by hypothalamic oxytocin signaling.

**Supplementary Information:**

The online version contains supplementary material available at 10.1186/s12915-022-01405-0.

## Background

In the vertebrate visual system, the retina initiates visual information processing. While retinal neurons use synaptic neurotransmitters, including glutamate, gamma-aminobutyric acid (GABA), and glycine, to encode and transmit visual information, neuromodulators, e.g. dopamine, nitric oxide, orexin, neuropeptide Y, and other neuropeptides, are also pivotal in the retina for visual information processing via modulating neuronal activity and synaptic transmission [1–5]. Dysfunction of neuromodulatory systems in the retina may result in retinal diseases, including diabetic retinopathy and glaucoma [6–9]. Hence, deciphering the expression and function of neuromodulator-related signals in the retina will promote our understanding of the retinal visual system.

Oxytocin is a well-known neurohormone for its function in labor induction and lactation. Oxytocin also acts as a neuropeptide in modulating diverse brain functions, including social, maternal, and emotional behaviors [10–12]. Endogenous oxytocin is synthesized and released by oxytocin neurons, which are mainly distributed in the paraventricular nucleus of the hypothalamus (PVN) and supraoptic nucleus (SON). Oxytocin modulates neuronal activity and synaptic transmission via activating the G_q_ protein-coupled oxytocin receptors (OxtRs), which are broadly expressed in mouse brain areas, including the visual-related brain regions [13, 14]. In the mouse primary visual cortex, oxytocin signaling mediates the experience-dependent cortical development [15] and also modulates spontaneous activity patterns in the developing visual cortex [16]. Few studies have investigated the involvement of oxytocin signaling in visual information transmission and processing. In the primate visual pathway, OxtRs have been detected in the superior colliculus, pulvinar, and primary visual cortex to modulate gaze direction and attention [17]. Oxytocin has also been detected in the retina of rats, bovines, and humans, and the retinal oxytocin concentration is synchronized with the day/night cycle [18, 19], but the expression and function of oxytocin signal in the retina are still largely unknown. Recent studies observed the OxtR expression in the retinal pigment epithelium (RPE) of humans and rhesus, and they inferred that oxytocin signaling played an important role in the RPE-photoreceptor communication [20, 21]. However, whether OxtRs are functionally expressed in retinal neurons and the role of retinal oxytocin signaling in visual information processing remains largely unknown.

In the mammalian retina, dopamine (DA), released by DA amacrine cells, has multiple neuromodulatory roles in visual functions related to light and contrast adaptation, visual acuity, and circadian rhythmicity [1]. In addition to integrating light inputs from classic photoreceptors and intrinsically photosensitive retinal ganglion cells [22], DA amacrine cells also express some neuromodulator receptors [23] and can be modulated by some neuropeptides, including orexin and neuropeptide Y [5, 24]. Oxytocin signaling regulates the DA system at different levels, including directly modulating DA neuronal activity and DA release via OxtR in the brain [25–28]. It is unclear whether oxytocin and DA also form interacted neuromodulatory networks to precisely regulate visual information processing in the retina.

In the present study, we demonstrated the expression of OxtRs in mouse retinal neurons by combining fluorescent in situ hybridization (FISH), Western blot (WB), and Oxtr-Cre; Ai3 transgenic mouse line. Our study further systematically investigated the identity of OxtR neurons by immunofluorescence staining with different retinal neuronal markers and explored the possible functions of retinal OxtR expression in visual information processing via electroretinographic (ERG) recording together with pharmacological methods.

## Results

### OxtRs are expressed in mouse retinal INL and GCL

**Fig. 1:**
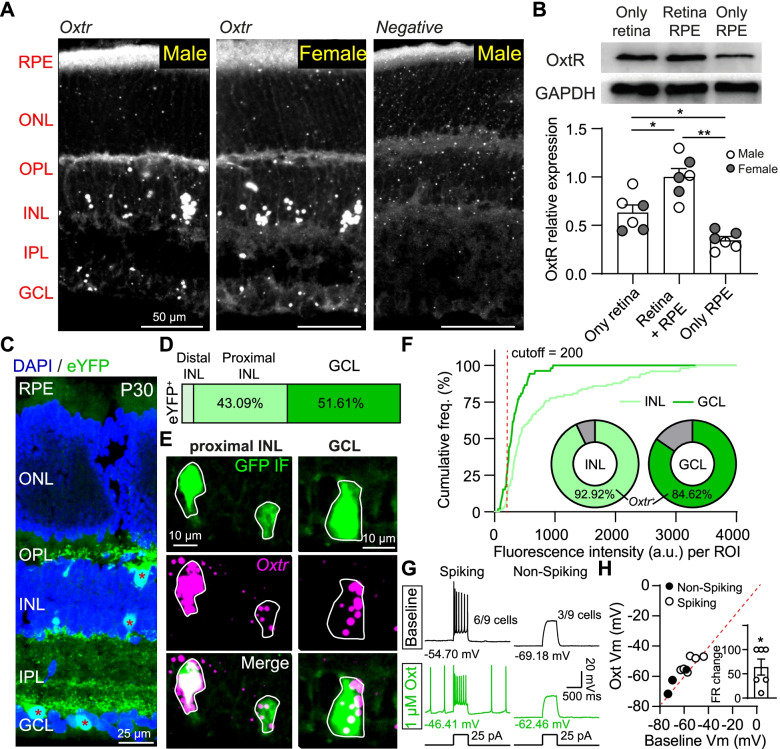
*Oxtr* mRNA and OxtR protein are expressed in mouse retinal INL and GCL. **A** Fluorescence in situ hybridization (FISH) images of *Oxtr* in male (left) and female (middle) mouse retinas. No signal was detected in the *negative* probe group (right). **B** OxtR protein is expressed in mouse retina and RPE. Top: OxtR protein level expression in the only retina, both retina and RPE, and only RPE of one mouse, determined by the Western blot assay. Bottom: summary of relative OxtR protein levels. *p < 0.05, **p < 0.01, *n* = 6 mice, including 3 males and 3 females, one-way ANOVA with Holm-Sidak post hoc tests. **C** An example image showing eYFP^+^ neurons in the retinal section in Oxtr-Cre; Ai3 mouse. **D** Summary of the proportion of eYFP^+^ neurons in retinal distal INL, proximal INL, and GCL. *n* = 622 eYFP^+^ neurons from 2 male and 2 female mice. **E** Confocal images showing the co-localization of eYFP^+^ neuron (GFP IF, green) and *Oxtr* mRNA puncta (magenta) in the proximal INL and GCL. **F** Summary of *Oxtr* mRNA fluorescence intensity in eYFP^+^ neurons. *n* = 99 and 52 eYFP^+^ neurons for proximal INL and GCL from 2 mice (1 male and 1 female), respectively. Insets indicate the ratios of eYFP^+^ neurons with *Oxtr* fluorescence intensity higher than 200 a.u. **G** The response traces of one Spiking eYFP^+^ neuron (left) and one non-spiking eYFP^+^ neuron (right) in the GCL before (top) and during (bottom) application of 1 μM oxytocin when blocking glutamatergic, GABAergic, and glycinergic synaptic neurotransmissions. **H** Summary of oxytocin-induced resting membrane potential change. *p < 0.05, *n* = 9 neurons, paired *t*-test. Inset indicates the firing rate (FR) increment of the spiking eYFP^+^ neuron. *p < 0.05, *n* = 6 neurons, one sample *t*-test

Oxytocin modulates neuronal activity via OxtRs, and there is only one type of OxtRs in the central nervous system [29]. To investigate the possibility of OxtR expression in retinal neurons, we firstly detected the *Oxtr* mRNA expression in adult mouse retina (~ 6 weeks old) with the fluorescence in situ hybridization (FISH) technique. As shown in Fig. 1A, *Oxtr*^+^ puncta were observed in both male and female mouse retinas, especially with a dense distribution in the inner nuclear layer (INL) and ganglion cell layer (GCL) (Fig. 1A), and the *Oxtr* fluorescence intensity of female and male mice in the INL and GCL was similar (Additional file 1: Fig. S1A). The expression of OxtR protein in mouse retina was further investigated with the Western blot (WB) assay (Fig. 1B and Additional file 1: Fig. S1B). Compared with OxtR mainly expressed in the RPE of humans and rhesus [20], we observed a higher expression of OxtR in adult mouse retina than in the RPE in a sex-invariant way (OxtR relative expression in both retina and RPE: 1.00 ± 0.2163; only in the retina: 0.6326 ± 0.1893; only in the RPE: 0.3474 ± 0.0894; *n* = 6 mice, including 3 males and 3 females, *p* < 0.001, one-way ANOVA with Holm-Sidak post hoc tests; Fig. 1B).

To further determine the expression of OxtR in retinal neurons, the Oxtr-Cre transgenic mouse line [30], which has Cre recombinase expression under the control of the endogenous OxtR promotor, was crossed with the Ai3 mouse line to investigate the distribution of OxtR neurons in the mouse retina. eYFP^+^ neurons were spaced across the whole retinal region with a denser expression close to the optic disk (Additional file 1: Fig. S1C) but only distributed in the INL and GCL (Fig. 1C), which is consistent with the expression of *Oxtr* mRNA (Fig. 1A). More than 90% of eYFP^+^ neurons were observed in the proximal INL and GCL without the sex difference, and ~ 5% were located at distal INL (Fig. 1D and Additional file 1: Fig. S1E). We verified the reliability of this transgenic mouse line to label OxtR retinal neurons by FISH and immunostaining (Fig. 1D). None of the eYFP^+^ neurons in the distal INL is co-localized with the *Oxtr* mRNA puncta (Additional file 1: Fig. S1D), but most of the eYFP^+^ neurons in the proximal INL and GCL co-express *Oxtr* mRNA (proximal INL: ~ 92.93%; GCL: ~ 84.62% from 2 mice; Fig. 1E, F). We further tested the functional regulation of oxytocin on retinal eYFP^+^ neurons with whole-cell current-clamp recording, and 1 μM oxytocin was used following previous studies [15, 16, 25]. After blocking glutamatergic, GABAergic, and glycinergic neurotransmissions by a cocktail (30 μM D-AP5, 40 μM DNQX, 50 μM L-AP4, 2 μM ACET, 10 μM bicuculline, 10 μM TPMPA,10 μM strychnine) [2, 31], eYFP^+^ neurons in the GCL with whole-mount retinal preparations were recorded. A total of 9 eYFP^+^ neurons in the GCL from 4 mouse retinas were recorded, 3/9 neurons did not exhibit action potential (non-spiking neuron), and 6/9 neurons had spontaneous or current injection-induced spikes (spiking neuron) (Fig. 1G and Additional file 1: Fig. S1F). The activities of all recorded neurons were depolarized by the oxytocin application, quantified by the resting membrane potential (baseline Vm: − 58.61 ± 3.01 mV; Oxt Vm: − 55.40 ± 2.70 mV; *p* = 0.035, *n* = 9 neurons, paired *t*-test) and firing rate change for the spiking neurons (FR change: 64.33% ± 16.47%, *p* = 0.011, *n* = 6 neurons, one sample *t*-test) (Fig. 1H and Additional file 1: Fig. S1F). Together, these results suggest that OxtR mRNA and protein are expressed in mouse retinal neurons in both INL and GCL.

### eYFP^+^ neurons in Oxtr-Cre; Ai3 mice are mainly retinal amacrine cells

Retinal neurons in the INL are classified into horizontal cells (HCs), bipolar cells (BCs), and amacrine cells (ACs). Retinal GCL is composed of ganglion cells (GCs) and displaced ACs. Different retinal neurons have distinct roles in visual information transmission and processing [32]. To determine which retinal neuron(s) may express OxtR, a series of biomarkers for specific subtypes of retinal neurons were used to determine the co-localization of eYFP^+^ neurons in Oxtr-Cre; Ai3 mice with different types of retinal neurons.

**Fig. 2:**
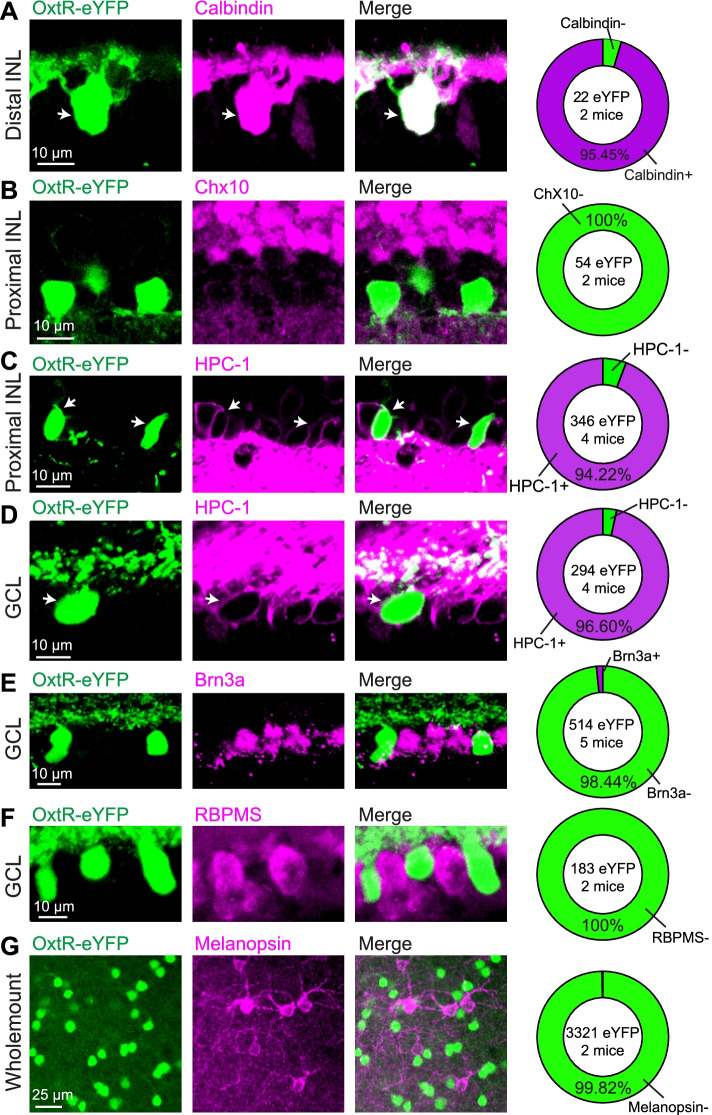
Most of the retinal eYFP^+^ neurons in Oxtr-Cre; Ai3 adult mice (OxtR-eYFP) are retinal amacrine cells. The retinal sections from Oxtr-Cre; Ai3 mice were immunostained with the horizontal cell marker—calbindin (**A**); bipolar cell marker—Chx10 (**B**); amacrine cell marker—HPC-1 in INL (**C**) and GCL (**D**); ganglion cell markers—Brn3a (**E**) and RBPMS (**F**); and ipRGC marker—Melanopsin (UF008) (**G**). Arrows indicate OxtR neurons that are also immunopositive to the cell type-specific marker. Right panels: summary results about the co-localization of OxtR-eYFP^+^ neurons with each cell type

Since eYFP^+^ neurons were observed in both proximal and distal INL, we first explored the co-localizations of eYFP^+^ neurons with HCs, BCs, and ACs (Fig. 2). As shown in Fig. 2A, almost all of the eYFP^+^ neurons in the distal INL are immunoreactive to Calbindin (95.45%, 21/22 eYFP^+^ neurons from 2 mice), which suggests that these neurons are HCs. Chx10 is the biomarker for BCs, and no overlap was observed between eYFP^+^ neurons and Chx10^+^ neurons in this study (0%, 0/54 eYFP^+^ neurons from 2 mice; Fig. 2B). In the proximal INL, more than 90% of eYFP^+^ neurons examined were co-localized with the HPC-1, an AC marker (94.22%, 326/346 eYFP^+^ neurons from 4 mice; Fig. 2C). Then, the identities of eYFP^+^ neurons in the GCL were investigated. More than 95% of eYFP^+^ neurons in GCL were immunopositive to HPC-1 (96.60%, 284/294 eYFP^+^ neurons from 4 mice; Fig. 2D), but few exhibited the immunoreactivity for GC markers—Brn3a (1.56%, 8/514 eYFP^+^ neurons from 5 mice) or RBPMS (0%, 0/183 eYFP^+^ neurons from 2 mice; Fig. 2F and Additional file 1: Fig. S2A). In addition to the regular RGCs, intrinsically photosensitive retinal ganglion cells (ipRGCs), which respond to light in the absence of photoreceptor input, exist in the retina [33]. Similar to the regular RGCs, few eYFP^+^ neurons in the whole-mount retina were observed to be immunopositive to the melanopsin (UF008), an ipRGC marker (0.18%, 6/3321 eYFP^+^ neurons from 2 mice; Fig. 2G and Additional file 1: Fig. S2B).

These immunoreactive results suggest that retinal eYFP^+^ neurons from Oxtr-Cre: Ai3 mice are mainly retinal HCs or ACs (Fig. 2), which are well-known inhibitory interneurons and provide lateral inhibition to regulate vertical excitatory circuits [34]. Since our FISH results showed that eYFP^+^ neurons in the proximal INL and GCL, but not in the distal INL, express *Oxtr* mRNAs in adult mice (Fig. 1 and Additional file 1: Fig. S1), we speculate that oxytocin may target OxtR expressed in the ACs to modulate visual information processing and transmission in the adult animal.

### eYFP^+^ neurons in Oxtr-Cre; Ai3 mice are mainly GABAergic amacrine cells

Retinal ACs are classified into more than 60 distinct subtypes according to their morphologies and molecular expression [23, 35]. The extensive diversity of ACs is important for modulating different visual information transmitted from BCs to RGCs [23, 36]. As eYFP^+^ neurons in Oxtr-Cre; Ai3 mice are mainly amacrine cells, we further investigated the subtype(s) of these eYFP^+^ neurons.

**Fig. 3:**
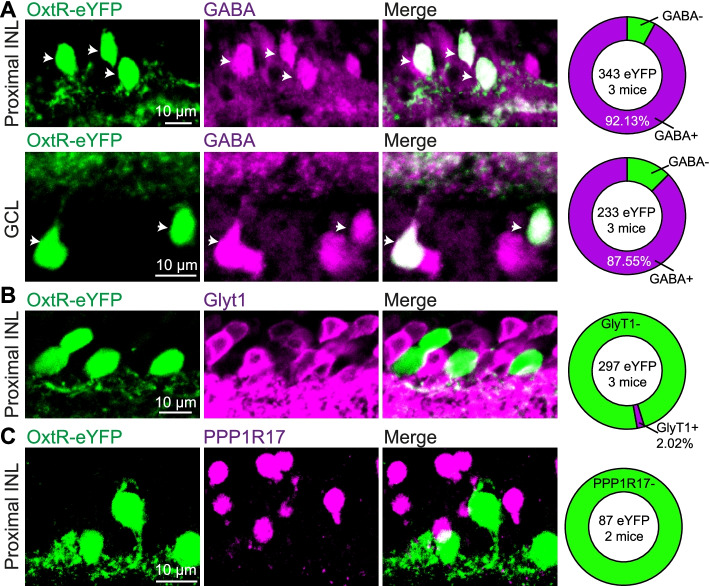
Most of the retinal eYFP^+^ neurons in Oxtr-Cre; Ai3 mice (OxtR-eYFP) are GABAergic amacrine cells. The retinal sections from Oxtr-Cre; Ai3 mice were immunostained for GABAergic cell markers—GABA in INL (**A**, top) and GCL (**A**, bottom); glycinergic cell marker—GlyT1 (**B**); and nGnG cell marker—PPP1R17 (**C**). Arrows indicate OxtR-eYFP^+^ neurons that are also immunopositive. Right column: summary results about the co-localization of OxtR-eYFP^+^ neurons with each cell type

Since the majority of ACs use GABA or glycine as neurotransmitters, we first investigated which neurotransmitter(s) these eYFP^+^ ACs in Oxtr-Cre; Ai3 mice may release. Double-labeling experiments demonstrated that about 90% of eYFP^+^ neurons are labeled by the GABAergic neuron markers—GABA in both INL (92.13%, 316/343 eYFP^+^ neurons from 3 mice) and GCL (87.55%, 204/233 eYFP^+^ neurons from 3 mice) (Fig. 3A), and GAD65/67 (glutamic acid decarboxylase 65/67) in INL (93.10%, 108/116 eYFP^+^ neurons from 2 mice) and GCL (88.39%, 137/155 eYFP^+^ neurons from 2 mice) (Additional file 1: Fig. S3A). However, few eYFP^+^ neurons were immunolabeled by glycinergic neuronal marker—glycine transporter 1 (GlyT1) (2.02%, 6/297 eYFP^+^ neurons from 3 mice; Fig. 3B and Additional file 1: Fig. S3B). In addition to GABAergic and glycinergic ACs, some ACs express neither GABA nor glycine (nGnG ACs), which are identified by a specific molecular marker—PPP1R17 [37, 38]. In our study, none of the eYFP^+^ neurons are co-labeled with PPP1R17 (0%, 0/87 eYFP^+^ neurons from 2 mice; Fig. 3C). Together, these results indicate that eYFP^+^ ACs in both INL and GCL mainly belong to the GABAergic AC subgroup.

**Fig. 4:**
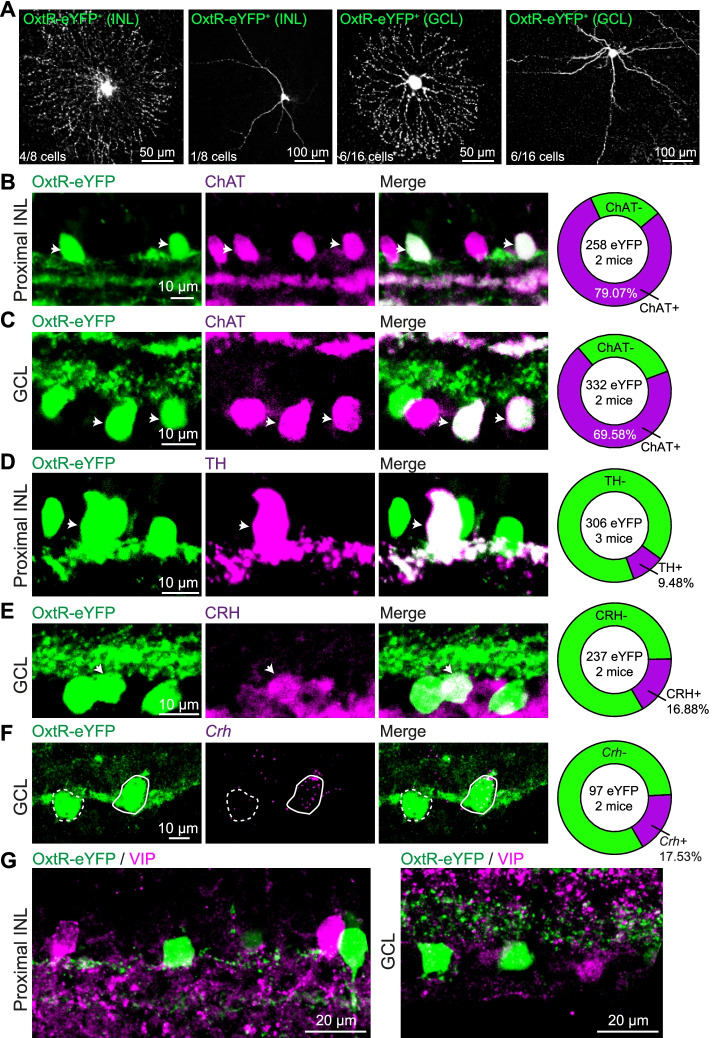
eYFP^+^ neurons in Oxtr-Cre; Ai3 mice are co-localized with retinal ChAT, DA, and CRH ACs. **A** Morphological examples of OxtR-eYFP^+^ neurons in Oxtr-Cre; Ai3 mice. **B**, **C** Co-localization of eYFP^+^ neurons with the ChAT immunopositive neurons in the proximal INL and GCL, respectively. **D** Retinal sections from Oxtr-Cre; Ai3 mice were immunostained for the DA cell marker—tyrosine hydroxylase (TH). **E**, **F** Immunostaining shows that some eYFP^+^ neurons are positive for CRH and *Crh* mRNA, respectively. **G** Example images show no overlap between eYFP^+^ neurons and VIP neurons in the INL (left) and GCL (right)

GABAergic ACs are divided into more than 35 subtypes based on their gene expression, including neurotransmitter, neuromodulator, and neuromodulator receptor-related genes [23], so the subtypes of GABAergic ACs colocalized with eYFP^+^ neuron in Oxtr-Cre; Ai3 mice were further investigated. We first visualized the morphologies of eYFP^+^ neurons by loading biocytin into the eYFP^+^ neurons in the INL and GCL, and most of the labeled neurons are medium-field and wide-field (10/24 medium-field cells and 7/24 wide-field cells; Fig. 4A). According to the neuronal morphologies, we speculated that eYFP^+^ neurons may include the cholinergic (ChAT, or starburst) AC, dopaminergic (DA) AC, corticotrophin-releasing hormone (CRH) AC, and vasoactive intestinal peptide (VIP) AC [23, 39]. Biomarkers of these ACs were used for further investigation.

In the proximal INL, ~ 80% of eYFP^+^ neurons from Oxtr-Cre; Ai3 mice are ChAT immunopositive (79.07%, 204/258 eYFP^+^ neurons from 2 mice; Fig. 4B), and the overlap ratio is about 69.58% in the GCL (231/332 eYFP^+^ neurons from 2 mice; Fig. 4C). DA ACs are wide-field AC and distributed in the proximal INL, and they are recognized by the large soma and specifically immunolabeled by tyrosine hydroxylase (TH) antibody. We observed that ~ 10% of eYFP^+^ neurons in the proximal INL are immunopositive to TH (9.48%, 29/306 eYFP^+^ neurons from 3 mice; Fig. 4D). CRH ACs are also wide-field but mainly distributed in the GCL [23, 39]. About 16.88% of eYFP^+^ neurons in the GCL are CRH immunopositive (40/306 eYFP^+^ neurons from 2 mice; Fig. 4E). We further confirmed that 17.53% of eYFP^+^ neurons in the GCL express *Crh* mRNA (17/97 eYFP^+^ neurons from 2 mice; Fig. 4F), which is consistent with the immunolabeling result. VIP ACs contain both medium-field and wide-field ACs and are distributed in both INL and GCL [40], but in this study, none of the eYFP^+^ neurons was observed to be co-localized with VIP immunolabeled neurons in INL and GCL (Fig. 4G). In addition, we analyzed the distribution of neurites stratifying in the retinal IPL by evenly dividing IPL into 5 strata [41], and the average fluorescence intensity in each stratum was measured to represent the neurite stratifications of retinal eYFP^+^ neurons [41]. We observed that the fluorescence-labeled dendrites were mainly clustered in IPL s1, s2 s4, and s5, but few in s3 (Additional file 1: Fig. S4A-B), which are consistent with the lamination of DA, ChAT, and CRH AC dendrites. Together, these results suggest that OxtRs are mainly expressed in retinal DA, ChAT, and CRH ACs in both INL and GCL in adult mice (Additional file 1: Fig. S4C).

### Retinal DA ACs extensively express *Oxtr* and are activated by oxytocin

**Fig. 5:**
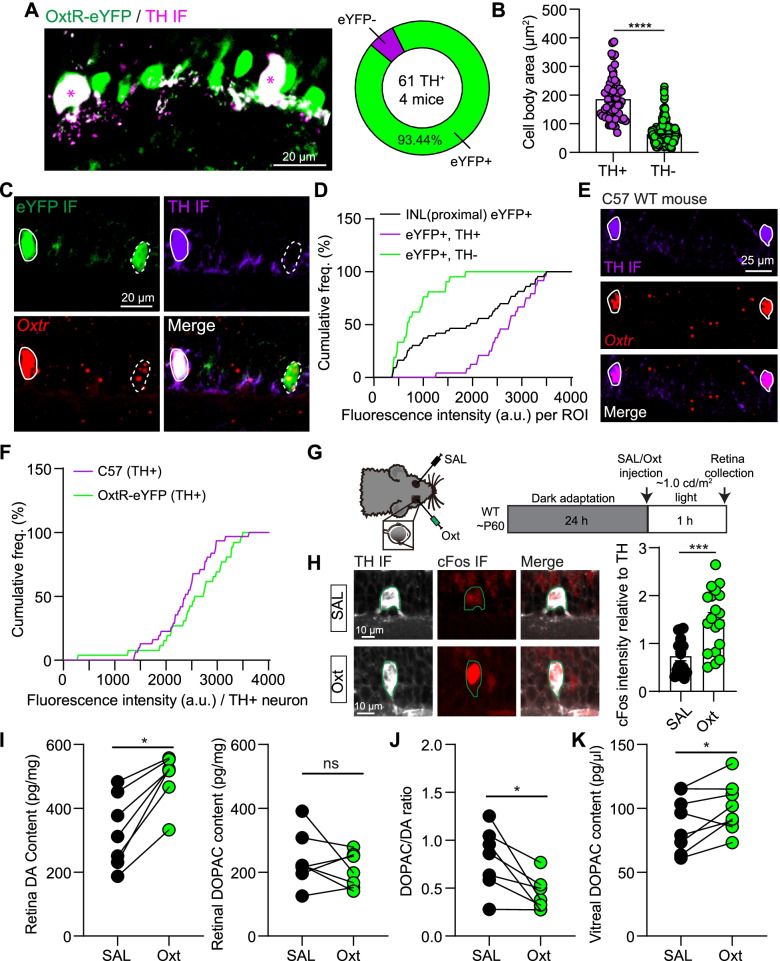
Retinal DA ACs express a high level of *Oxtr* mRNAs and are activated by oxytocin. **A** Left: an example image showing retinal DA neurons (TH IF) co-localized with OxtR-eYFP neurons. Right: summary result showing that more than 93% of TH^+^ neurons co-localized with OxtR-eYFP neurons. *n* = 61 TH^+^ neurons from 4 mice. **B** Statistical results about soma area of eYFP^+^, TH^+^ ACs and eYFP^+^, TH^−^ ACs. *n* = 57 TH^+^ and 310 TH^−^ of eYFP^+^ neurons from 4 mice, *p* < 0.0001, Mann–Whitney test. **C** Confocal images showing co-localization of eYFP^+^ neuron (GFP IF), TH^+^ neuron, and *Oxtr* mRNA puncta. White solid and dashed circles indicate eYFP^+^, TH^+^ and eYFP^+^, TH^−^ neurons, respectively. **D** Distribution of *Oxtr* fluorescence intensity in retinal eYFP^+^ neurons, eYFP^+^, TH^+^ neurons, and eYFP^+^, TH^−^ neurons. *n* = 24 and 21 eYFP^+^ neurons for TH^+^ and TH^−^ from 2 mice, respectively. **E** Confocal images showing co-localization of TH^+^ neuron and *Oxtr* mRNA puncta from a C57 WT mouse. Circles indicate TH^+^ neurons. **F** Quantitative analysis of *Oxtr* fluorescence intensity in DA ACs of WT mice and Oxtr-Cre; Ai3 mice. *n* = 32 and 24 TH^+^ neurons from 2 mice for WT mice and Oxtr-Cre; Ai3 mice, respectively. **G** Schematic of intravitreal injection (left) and experimental protocol (right). **H** Left: examples of cFos expression in TH neurons with saline (SAL, top) and oxytocin (Oxt, bottom) application. Right: summary about cFos intensity relative to TH signal in TH^+^ neurons in SAL and Oxt conditions. ***p < 0.001, Mann–Whitney test, *n* = 17 and 19 TH^+^ neurons for the SAL and Oxt groups from 2 mice, respectively. **I** Summaries of retinal DA (left) and DOPAC (right) concentrations with intravitreal injecting SAL and Oxt. *p < 0.05, Wilcoxon matched-pairs signed-rank test, *n* = 7 mice. **J** Summary of the ratio between retinal DOPAC and DA with intravitreal injecting SAL and Oxt. *p < 0.05, Wilcoxon matched-pairs signed-rank test, *n* = 7 mice. **K** Summary of vitreal DOPAC concentration with intravitreal injecting SAL and Oxt. *p < 0.05, paired *t*-test, *n* = 8 mice

We further investigated the proportion of retinal eYFP^+^ neurons in different retinal ACs in Oxtr-Cre; Ai3 mice. Different from the ChAT and CRH ACs (Additional file 1: Fig. S5A), almost all of the retinal DA ACs are co-localized with eYFP^+^ neurons in Oxtr-Cre; Ai3 mice (93.44%, 57 TH^+^, eYFP^+^/61 TH^+^ neurons from 4 mice; Fig. 5A). Therefore, though only ~ 10% of eYFP^+^ neurons in Oxtr-Cre; Ai3 mice are TH^+^, more than 90% of TH^+^ ACs are OxtR-eYFP^+^. Consistent with the large soma size of DA ACs, the body area of double-labeled ACs is significantly larger than eYFP^+^, TH^−^ ACs (eYFP^+^, TH^+^: 186.40 ± 10.32 μm^2^; eYFP^+^, TH^−^: 63.64 ± 1.80 μm^2^; *p* < 0.0001, *n* = 57 for eYFP^+^, TH^+^ and 310 for eYFP^+^, TH^−^, Mann–Whitney test) (Fig. 5B). Surprisingly, combining FISH and TH immunostaining, we observed that TH^+^ ACs, co-labeled with eYFP signal in Oxtr-Cre; Ai3 mice, extensively expressed more *Oxtr* fluorescence than eYFP^+^, TH^−^ ACs (eYFP^+^, TH^+^: 2678.0 ± 115.0 a.u.; eYFP^+^, TH^−^: 829.5 ± 94.83 a.u. *n* = 24 and 21 for TH^+^ and TH^−^, respectively; unpaired *t*-test, *p* < 0.0001) (Fig. 5C, D. Negative control in Additional file 1: Fig. S5B).

To exclude the possibility of Cre knockin-induced abnormal expression of *Oxtr* mRNA, we investigated the expression of *Oxtr* mRNA in retinal DA neurons of C57BL/6 mice. Consistent with the Oxtr-Cre; Ai3 transgenic mice, retinal TH^+^ neurons are *Oxtr*^+^ (32/32 TH^+^ neurons from 2 mice), and the fluorescence intensity of *Oxtr*^+^ signal expressed in the soma of TH^+^ neurons of C57BL/6 mice were similar with that of Oxtr-Cre; Ai3 mice (C57, TH^+^: 2369.0 ± 97.39 a.u.; OxtR-eYFP, TH^+^: 2678.0 ± 115.0 a.u. *n* = 32 and 24 neurons for C57 and OxtR-eYFP mice, respectively; unpaired *t*-test, *p* = 0.2150) (Fig. 5E, F).

DA ACs are the releasing source of retinal DA, so we tested the effect of oxytocin on the activity of DA ACs and DA release in the mouse retina. Under the dim light environment (~ 1.0 cd/m^2^), intravitreal injection of oxytocin (1 μl, 1 mM) significantly increased the cFos intensity, an immediate early gene that is expressed within neurons following depolarization [22], of TH^+^ neurons in the INL (cFos intensity relative to TH in SAL: 0.7353 ± 0.0922 a.u.; in Oxt: 1.502 ± 0.1465 a.u. *p* < 0.001, Mann–Whitney test, *n* = 17 and 19 TH^+^ neurons from 2 mice, respectively) (Fig. 5G, H). Activating retinal DA neurons will increase DA release in the retina. In support of the increased activity of retinal DA neurons, intravitreal application of oxytocin elevated retinal DA level under a dim light environment (DA in SAL: 327.6 ± 42.88 pg/mg; DA in Oxt: 496.2 ± 29.48 pg/mg. *p* < 0.05, Wilcoxon matched-pairs signed-rank test, *n* = 7 mice) but had no significant effect on DA metabolites—DOPAC and HVA (Fig. 5I). The ratio between DOPAC and DA was significantly reduced by oxytocin (Fig. 5J). Even under the dark environment, oxytocin application also significantly increased retinal DA concentration but not the DOPAC or HVA (Additional file 1: Fig. S5C). The level of vitreal DOPAC is a robust indicator of retinal DA release [42, 43], and oxytocin application significantly increased the vitreal DOPAC level in a sex-invariant manner (vitreal DOPAC in SAL: 88.48 ± 7.83 pg/μl; vitreal DOPAC in Oxt: 100.40 ± 6.91 pg/μl; *p* < 0.05, paired *t*-test, *n* = 8 mice) (Fig. 5K and Additional file 1: Fig. S5D). Together, these results indicate that oxytocin can increase retinal DA neuronal activity and elevate retinal DA release.

### Oxytocin reduces the activity of retinal bipolar cells via oxytocin and DA receptors

**Fig. 6:**
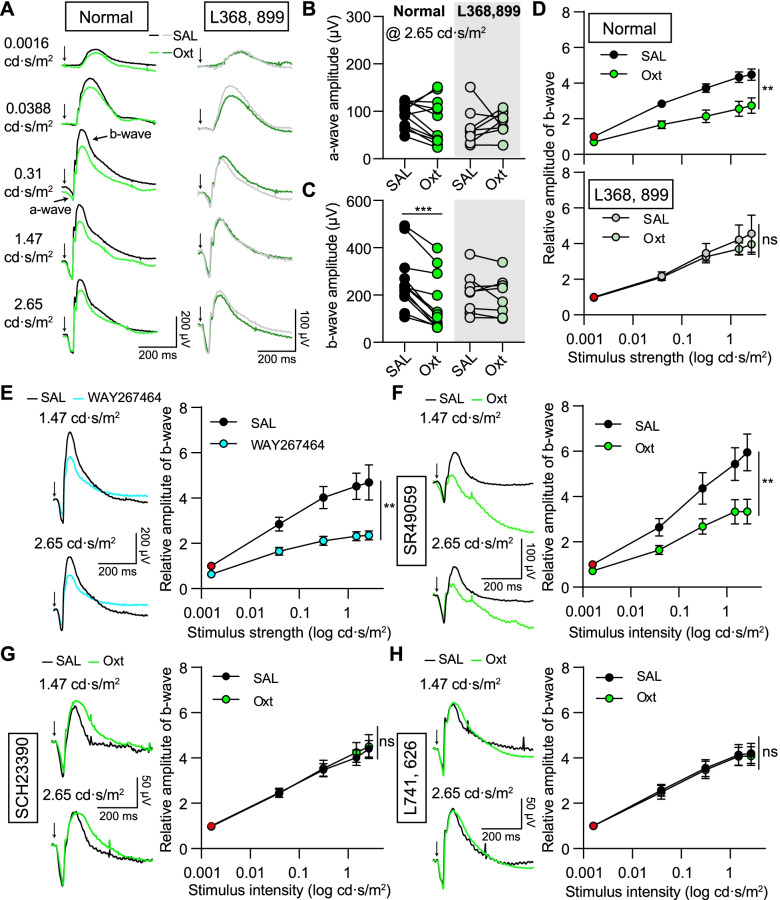
Oxytocin reduces ERG b-wave amplitude via OxtR and DA receptors. **A** Representative ERG recordings from animals with oxytocin (Oxt) injected into one eye, as compared with the recordings from the fellow eye with saline (SAL) injection. Left: normal condition; right: pre-treated with L368, 899. **B** Oxt has no significant effect on a-wave amplitude with light stimulation intensity at 2.65 cd·s/m^2^ in normal and L368, 899 pre-treated conditions. For normal condition, *n* = 12 mice, *p* = 0.1598. For L368, 899 treated condition, *n* = 8 mice, *p* = 0.6636, paired *t*-test. **C** Same as **B**, but for ERG b-wave amplitude. ***p < 0.001, Wilcoxon matched-pairs signed-rank test. **D** Effect of Oxt on b-wave relative amplitude in normal (top) and L368, 899 treated (bottom) conditions. ERG b-wave amplitudes were normalized to b-wave amplitude recorded from the eye with SAL injection and the stimulation intensity at 0.0016 cd·s/m^2^, labeled with the red circle. **p < 0.01, two-way ANOVA test. **E** Left: representative ERG recordings from one mouse with WAY267464 injected into one eye and SAL injected into the fellow eye with light stimulation intensity at 1.47 cd·s/m^2^ (top) and 2.65 cd·s/m^2^ (Bottom). Right: effect of WAY267464 on b-wave relative amplitude. *n* = 7 mice, **p < 0.01, two-way ANOVA test. **F** Left: representative ERG traces from one mouse pre-treated with SR49059 and then injected Oxt into one eye and SAL into the fellow eye. Right: effect of Oxt on ERG b-wave relative amplitude when pre-treated with SR49059. *n* = 9 mice, **p < 0.01, two-way ANOVA test. **G**–**H** Same as **F**, but for the mice pre-treated with D1 receptor antagonist—SCH23390 (**G**)—and D2 receptor antagonist—L741, 626 (**H**). *n* = 8 mice for SCH23390 experiment and L741, 626 experiment, separately. *p* > 0.05, two-way ANOVA test

Retinal DA has multiple functions, including adjusting retinal light sensitivity via regulating bipolar cells [44, 45]; we further investigated the functional effect of oxytocin on retinal visual information processing by measuring the b-wave component of the ERG signal, which is used to quantify the activity of bipolar cells [46, 47]. For the dark-adapted mice, compared with the intravitreal saline injection into one eye, oxytocin (1 mM, 1 μl) injection into the other eye significantly decreased the amplitude of b-wave in response to the flashlight stimulations (for the b-wave amplitude induced by 2.65 cd·s/m^2^ light stimulation, SAL: 259.4 ± 34.82 μV; oxytocin: 160.1 ± 34.14 μV; *n* = 12 mice, *p* < 0.001, Wilcoxon matched-pairs signed-rank test; Fig. 6A–D). The oxytocin-induced b-wave amplitude reduction was consistent in males and females (Additional file 1: Fig. S6C-S6D). The amplitude of the a-wave, reflecting the activity of retinal photoreceptors [47], was not changed with the oxytocin application (Fig. 6A–D). We also investigated the effect of oxytocin at different concentrations, including 1 μM, 10 μM, 100 μM, and 500 μM, on the amplitude of ERG b-wave (Additional file 1: Fig. S6A). Intravitreal injection of 1 μM oxytocin still significantly reduced the ERG b-wave amplitude. The volume of mouse vitreous volume is ~ 4.5 μl [48], so the 1 μl 1 μM oxytocin would be diluted to be ~ 180 nM after intravitreal injection. Therefore, exogenous oxytocin at the nanomolar concentration is sufficient to reduce the amplitude of ERG b-waves. Consistent with the oxytocin injection, intravitreal injection of the specific and selective OxtR agonist WAY267464 also significantly weakened the amplitude of the b-wave (for the b-wave amplitude induced by 2.65 cd·s/m^2^ light stimulation, SAL: 327.1 ± 52.33 μV; WAY267464: 177.5 ± 30.90 μV; *n* = 7 mice, *p* < 0.01, paired *t*-test; Fig. 6E and Additional file 1: Fig. S7A).

Oxytocin mainly binds to OxtR to modulate neuronal activity. For the mice intraperitoneally pre-treated with the OxtR antagonist—L368, 899—oxytocin (1 mM, 1 μl) intravitreal injection had little influence on the amplitude of b-wave (for the b-wave amplitude induced by 2.65 cd·s/m^2^ light stimulation, SAL: 209.3 ± 30.77 μV; oxytocin: 196.2 ± 29.42 μV; *n* = 8 mice, *p* = 0.3936, paired *t*-test; Fig. 6A–E). Intravitreal injecting L368, 899 (500 μM, 1 μl) had little influence on the b-wave amplitude (Additional file 1: Fig. S6B). In addition to OxtR, oxytocin may also activate vasopressin receptor 1A (V1AR) [25, 49]. However, after blocking V1AR with SR49059, oxytocin still significantly reduced the amplitude of ERG b-wave (Fig. 6F and Additional file 1: Fig. S7B). Retinal DA is reported to modulate the activity of bipolar cells and ERG b-wave amplitude via DA receptors, especially the D1 receptor [50–53]. To establish the involvement of DA signaling, DA D1 receptor antagonist—SCH23390—was intraperitoneally injected ~ 30 min before oxytocin application, and the effect of oxytocin-induced b-wave amplitude reduction disappeared (Fig. 6G and Additional file 1: Fig. S7B). DA D2 receptors are mainly present in retinal photoreceptors and DA neurons [54, 55]. Oxytocin-induced b-wave amplitude reduction was also blocked by the pre-treatment of DA D2 receptor antagonist—L741, 626 (Fig. 6H and Additional file 1: Fig. S7B). The effect of blocking D2 receptors on retinal DA neurons may override the oxytocin-induced retinal DA change. Together, these results suggest that oxytocin suppresses the activity of bipolar cells via retinal OxtR and DA-related signals.

## Discussion

As an important neuropeptide, oxytocin has multiple important functions in the brain [11]. Though OxtR expression has been reported in the retinal pigment epithelium [20, 21], our study establishes that OxtRs are specifically expressed in mouse retinal GABAergic amacrine neurons, especially in DA, ChAT, and CRH ACs (Additional file 1: Fig. S4). By detecting retinal DA and vitreal DOPAC concentrations, evaluating cFos expression and measuring ERG, we provide compelling evidence that exogenous oxytocin is sufficient to regulate visual information transmission via modulating DA-related signal pathways. Therefore, our study suggests that the oxytocin signal is involved in visual information processing via the retinal GABAergic amacrine cell network, especially the retinal dopaminergic pathway.

A previous study has detected oxytocin content in rat, bovine, and human retina [18], and OxtR has been reported to be expressed in the RPE of the human and rhesus retinas [20]. In this study, we proved that OxtRs are functionally expressed in mouse retinal neurons in three ways. First, OxtR mRNAs were detected by fluorescence in situ hybridization, and we observed that *Oxtr*^+^ puncta are densely distributed in retinal INL and GCL. Second, we measured the OxtR protein expression in the retina. With the Oxtr-Cre; Ai3 transgenic mice, we found that OxtR-eYFP neurons are amacrine and displaced amacrine cells in the INL and GCL, and oxytocin application increased the activity of OxtR-eYFP neurons. Our electrophysiological recordings in GCL found that oxytocin increased the activities of both spiking and non-spiking neurons. Though OxtR-eYFP neurons are displaced amacrine cells in the GCL and starburst amacrine cells do not have action potentials in adult mice [56], some types of amacrine cells in this layer, including CRH amacrine neurons, are reported to fire action potentials [39]. Third, exogenous oxytocin application is sufficient to increase the activity of retinal DA neurons and elevate DA release via OxtR. cFos expression has been used to represent the activation of retinal DA amacrine cells [22]. Though OxtRs are mainly expressed in GABAergic ACs, which may release GABA to inhibit DA neurons, more than 90% of retinal DA neurons in mice express a high level of OxtR mRNA, and oxytocin is expected to directly depolarize the DA neurons via OxtRs. Intravitreal injection of oxytocin in nanomolar was also sufficient to reduce the amplitude of ERG b-wave via OxtRs and DA receptors.

A recent high-throughput single-cell RNA sequencing study suggests that some amacrine cells express neuropeptide genes, including *Oxt* [23]. However, oxytocin protein was not detected in mouse amacrine cells [20], so we infer that the retinal endogenous oxytocin may be from oxytocin neurons in the hypothalamus via blood circulation or direct projection [57]. The concentration of plasma oxytocin in adult mice is reported to be ~ 4 ng/ml [15]. Recent studies suggested the possibility of oxytocin passing the blood–brain barrier and found that oxytocin could be transported into the brain by the receptor for advanced glycation end-products (RAGE) on the brain capillary endothelial cells [58, 59]. RAGEs are also expressed in the retinal blood vessels [60], so oxytocin in blood circulation may pass the blood-retina barrier to regulate retinal functions. Since hypothalamic oxytocin release is changed with the external environment (e.g., enriched environment [15] and social touch [61, 62]) and internal physiology (e.g., parental caring [63, 64] and stressful state [65]), the processing and transmitting visual information in the retina may be regulated by animal states via hypothalamic oxytocin signaling.

Endogenous oxytocin signal plays pivotal roles in refining synaptic connections and promoting excitatory synaptic transmission in mouse sensory cortices, including the visual cortex, during development [15, 16]. In the visual cortex, OxtR is found to be expressed in the GABAergic interneurons to modulate the excitatory/inhibitory ratio to refine the developing circuits [16]. As OxtR is also expressed in the GABAergic interneurons in the retina, endogenous oxytocin signal in the retina may act to refine the retinal circuitry during the development. In humans, the role of endogenous oxytocin signals in social perceptual processes, such as perception of facial emotions and visual processing of infant faces [64], has been reported. Enriched social environment, social touch, parental caring, and parent-infant interaction are reported to increase oxytocin levels [15, 61–64]. Endogenous oxytocin signal in the retina may be involved in promoting social recognition and parental behavior via elevating visual acuity and detection, which requires to be investigated in the future.

In addition to directly regulating neuronal excitability, oxytocin plays a pivotal role in regulating neuronal synaptic transmission via OxtRs expressed in both presynaptic and postsynaptic membranes [13, 26, 66]. Though OxtRs are found to be expressed in several types of retinal GABA amacrine cells, we did not investigate whether oxytocin acts presynaptically or postsynaptically to modulate synaptic transmission in the retina. Amacrine cells bi-directionally communicate with bipolar cells and retinal ganglion cells in the INL to regulate visual information processing, so further studies about the synaptic mechanisms underlying oxytocinergic modulation in the retina will precisely uncover the function of oxytocin signal in modulating visual transmission in the retina.

Retinal amacrine cells are important retinal inhibitory interneurons, and amacrine cells are diverse in morphology, electrophysiology, and molecular expression, which are required for rendering the functional diversity and selectivity of RGCs [23, 32, 35]. Transcriptomic analyses showed that mouse retinal amacrine cells are classified into as many as 63 subtypes [23], and each subtype has its functions in encoding and processing visual information. Our study found that OxtRs are mainly expressed in three subtypes of GABAergic amacrine cells: ChAT, DA, and CRH amacrine cells. ChAT amacrine cells, also called starburst amacrine cells, serve as the key element in detecting visual stimulus movement [67]. The superior colliculus (SC) is involved in encoding moving visual stimuli [68–70], and PVN oxytocin neurons directly receive excitatory synaptic inputs from SC neurons [63]. Therefore, OxtR expression in ChAT amacrine cells may act as positive feedback to enhance the salience of moving stimuli, which is important for preying on and avoiding predators. Different from ChAT amacrine cells, almost all of the retinal DA neurons are OxtR-positive and express a high level of *Oxtr* mRNAs. In the retina, DA amacrine cells show a pronounced daily rhythmic DA production and release [1]. Secretion of oxytocin from the hypothalamus shows a diurnal circadian rhythmic pattern, and oxytocin content in the retina also exhibits a day/night rhythm [19]. In our study, oxytocin application in the retina significantly elevated the DA content. Since both DA and oxytocin contents are increased with light stimulation, the retinal oxytocin signal may be involved in regulating the activity of DA amacrine cells and rhythmic DA release. CRH amacrine cells are ON-pathway amacrine cells, and they converge with OFF-pathway amacrine cells to balance RGCs’ excitation [39]. In the brain, the oxytocin signal is also involved in regulating synaptic transmission to balance excitation and inhibition [15, 26]. Hence, OxtR/CRH double-positive amacrine cells may also act to regulate the synaptic inputs to RGCs to sculpt visual information processing.

OxtR is a Gq protein-coupled receptor, and activation of this receptor will increase neuronal excitability [29]. Since *Oxtr* is highly expressed in all of the DA amacrine cells, oxytocin application increased the cFos expression in DA neurons and elevated retinal DA release. In the mammalian retina, DA amacrine cells show a pronounced daily rhythmic DA production and release, which is important to maintain visual sensitivity from dim to bright ambient light conditions [1]. DA D1 and D2 receptors are differently expressed in the retina, with D1 receptors expressed in a broad range of retinal neurons, whereas D2 receptors are mainly in photoreceptors and DA neurons [55, 71]. Activating D1 receptors will increase the activity of GABA(A) receptors on dendrites of ON bipolar cells and also suppress voltage-gated calcium currents in some bipolar cells, which leads to a strong inhibition of the activity of bipolar cells and the reduction of ERG b-wave amplitude [52, 53]. Previous studies also reported that the application of exogenous DA and its agonist decreased the amplitude of the b-wave in the mammalian retina [51, 72]. In addition to oxytocinergic regulation of the visual system, the visual signal from the superior colliculus is also found to activate hypothalamic oxytocin neurons [63]. Therefore, the hypothalamic oxytocin signal and the visual signal may reciprocally facilitate to enhance visual sensitivity.

Though previous studies suggested that oxytocin system could act differently in males and females [13, 73–75], the neuronal properties and projections of PVN oxytocin neurons are found to be sex invariance in mice [76, 77], and the modulation function of oxytocin signal in visual cortex was reported to be similar in different genders [15, 16]. In our study, we compared the differences in retinal oxytocin signals between males and females in the following four aspects: (1) *Oxtr* fluorescence intensities in the INL and GCL are not significantly different between males and females (Additional file 1: Fig. S1A); (2) the numbers of OxtR-eYFP^+^ neurons from Oxtr-Cre; Ai3 mice in the INL and GCL did not exhibit sex difference (Additional file 1: Fig. S1E); (3) oxytocin-induced retinal DA release was similar in both male and female mice (Additional file 1: Fig. S5D); and (4) intravitreal injection of oxytocin significantly reduced the amplitude of ERG b-wave in both males and females, and the oxytocin-induced ERG b-wave change did not show significant sex difference (Additional file 1: Fig. S6C-S6D). Together, these results indicate that the expression and function of oxytocin signals in adult mouse retinas largely do not exhibit sex differences.

## Conclusions

In summary, our study finds that OxtR is functionally expressed in retinal GABAergic ACs, especially in DA subtypes. The possible functions of neuromodulators synthesized by retinal neurons have been extensively investigated [1, 5, 55, 78], our study indicates that neuropeptides synthesized and released from the brain may also involve in regulating retinal visual perception. Though our results suggest activation of OxtR is sufficient to regulate visual information processing, the physiological functions of endogenous oxytocin signal are worth to be investigated in the future. Previous studies found that endogenous oxytocin directly regulates midbrain dopamine neurons to promote pro-social behavior [25, 27], so our study expands the interactions between oxytocin and dopamine systems.

## Methods

### Mouse strains and genotyping

Animals were handled following the protocols approved by the Fudan University Animal Care and Use Committee. Mice were housed on a 12:12 light/dark cycle (8 AM light on and 8 PM light off) with ad libitum access to food and water. Both male and female rodents were used in this study. C57BL/6 mice were obtained from Shanghai Model Organisms Center. B6.Cg-Oxtr^*tm1.1(cre)Hze*^/J mice (Oxtr-Cre, #031303, Jackson Laboratory) were used for labeling OxtR-expressing neurons. The floxed eYFP (Ai3) reporter strain was crossed with Oxtr-Cre mice to visualize OxtR^+^ neurons. Mouse genotyping was conducted following standard procedures on the Jackson Lab websites.

### Tissue processing, immunohistochemistry, and imaging

Mice were anesthetized with isoflurane, and the eyes were removed quickly and dissected in 0.1 M phosphate-buffered saline (PBS). Isolated eye cups were fixed in 4% PFA (dissolved in 0.1 M PBS) at 4 °C for 6 h then dehydrated with 10% (w/v) sucrose solution (dissolved in 0.1 M PBS) for 2 h, 20% for 2 h, and 30% for 24 h in sequence. The dehydrated eyes were embedded in OCT and cut at a thickness of 15 μm using a cryostat (CM1950, Leica Microsystems). The sections were mounted onto Superfrost Plus slides (Thermo Fisher Scientific, Waltham, MA) and stored at − 80 °C.

For immunostaining to determine neuronal identity, tissues were rinsed with PBS and pretreated in 0.2% Triton-X100 for 1 h at room temperature (RT) then blocked with 0.05% Triton-X100 and 10% bovine serum albumin (BSA) in PBS for 2 h at RT. Tissues were then incubated with primary antibody solution in PBS with 0.2% Triton-X100 and 1% BSA for 2–3 days at 4 °C. After rinsing in PBS three times, the tissues were incubated with secondary antibody solution (goat anti-rabbit 488, 594, 647; goat anti-mouse 647, 1:800; goat anti-guinea pig 647, 1:800; goat anti-sheep 648, 1:800, Life Technologies; donkey anti-goat 594, 1:200, Jackson ImmunoResearch) in PBS for 2 h at RT then dried and covered under glycerol:TBS (3:1) with Hoechst 33,342 (1:1000, Thermo Fisher Scientific). The primary antibodies used in this study include mouse anti-HPC-1 (1:1000, S0664, Sigma), sheep anti-Chx10 (1:1000, ab16141, Abcam), mouse anti-Calbindin (1:1000, CB300, Swant), mouse anti-Brn3a (1:50, MAB1585, Sigma), guinea pig anti-RBPMS (1:500, 43,691, PhosphoSolutions), rabbit anti-Melanopsin (UF008, 1:10,000, AB-N39, ATS), rabbit anti-vasoactive intestinal peptide (VIP, 1:1000, 20,077, Immunostar), rabbit anti-GAD65 + GAD67 (1:1000, ab183999, Abcam), rabbit anti-GABA (1:1000, A2052, Sigma), rabbit anti-GlyT1 (1:1000, AGT-011, Alomone), rabbit anti-PPP1R17 (1:500, HPA047819, the Human Protein Atlas), goat anti-ChAT (1:800, ab144p, Millipore), rabbit anti-tyrosine hydroxylase (TH, 1:1000, ab152, Millipore), mouse anti-TH (1:1000, 22,941, Immunostar), and rabbit anti-CRH (1:100, ab8901, Abcam). The sections were imaged with an Olympus VS120 slide scanning microscope. Confocal images were acquired with a Nikon A1 confocal laser scanning microscope with a × 25 objective. Images were analyzed in ImageJ (FIJI).

### Quantitative fluorescence single-molecule in situ hybridization (smFISH)

The retina sections were prepared in the same way as used for immunohistochemistry. Samples were then processed according to the manufacturer’s instructions in the RNAscope Fluorescent Multiplex Assay manual (Advanced Cell Diagnostics, Newark, CA). After finishing smFISH, some samples were further stained with TH or GFP primary antibodies for 24 h at 4 °C then washed and incubated with secondary antibody. Samples were coverslipped with ProLong Gold antifade reagent with DAPI (Molecular Probes). The following probes were used in this study: *Oxtr* (C1, 406491), *Crh* (C1, 318931), and *EYFP* (C3, 312131). Sections were subsequently imaged with a Nikon A1 confocal laser scanning microscope with a × 25 objective lens, with 1 μm between adjacent *z*-sections. Probe omission or negative probes were carried out as control for every reaction.

smFISH images were analyzed as previously reported [25]. Every four adjacent *z*-stack images were combined. All channels were thresholded to remove background noise. Cellular regions of interest (ROIs) were defined using the GFP IF channel or TH IF channel to localize cell bodies. Since it is not easy to discriminate the single *Oxtr* punctum within ROIs, the cell in ROI was considered positive for *Oxtr* when the fluorescence intensity of *Oxtr* signal within the soma was more than 200 a.u. (based on the negative probe control). All counting experiments were conducted blinded to the experimental group.

### Western blot analysis

The mouse retinas were isolated under the microscope, and the retinal pigment epithelium (RPE) was isolated following a previous study [79]. Tissues were lysed with RIPA lysis buffer containing PMSF protease inhibitor (100:1), and total proteins were extracted and protein concentrations were quantified with a bicinchoninic acid (BCA) assay kit (Beyotime Biotech, China). The protein sample’s final concentration was 2 or 3 μg/μl by diluting with sample loading buffer and ddH_2_O. A total of 20 μg protein was loaded into the polyacrylamide gel and electrophoretically transferred to the polyvinylidene difluoride (PVDF) membrane. The PVDF membrane was blocked for 1 h in 5% non-fat powdered milk and then incubated with primary antibody (rabbit anti-OxtR, 1:2000, AB181077, Abcam) overnight at 4 °C. Mouse anti-GAPDH antibody (1:80,000, 60,004, Proteintech) was used as the control. After incubating with the primary antibody, the PVDF membrane was then rinsed in TBST three times, and incubated with HRP-goat anti-mouse IgG antibody (1:7000, SA00001-1, Proteintech) or HRP-goat anti-rabbit IgG antibody (1:6500, SA00001-2, Proteintech) at room temperature for 1 h. ECL Prime Western Blotting Detection Reagent was used for fluorescence detection by an Odyssey near-infrared imaging scanner (FluorChem E System, Protein Simple, USA). The analysis of images of blots was performed with the AlphaView SA software (Protein Simple, USA). The images of the original uncropped blots have been provided in Additional file 2.

### Electrophysiological recording

The retinas from Oxtr-Cre; Ai3 mice were prepared, and retinal neurons were recorded as previously described [2]. Briefly, mice were dark-adapted for at least 2-h and then anesthetized with 25% urethane (0.2 ml/100 g). The mouse retinas were dissected under dim red light in Ames’ medium (MilliporeSigma) and bubbled with 95% O_2_ and 5% CO_2_. The retina was placed in a recording chamber and perfused with oxygenated Ames’ solution at a rate of ~ 3 ml/min. eYFP-labeled neurons in the GCL were visualized using an IR-DIC microscopy. Current-clamp recordings were established with glass pipettes (5–7 MΩ) containing the following (in mM): 120 K-gluconate, 5 NaCl, 4 KCl, 10 HEPES, 2 EGTA, 4 Mg-ATP, 0.3 Na-GTP, and 7 Tris-phosphocreatine (pH was adjusted to 7.3); 30 μM D-AP5, 40 μM DNQX, 50 μM L-AP4, 2 μM ACET, 10 μM bicuculline, 10 μM TPMPA, and 10 μM strychnine are used to block NMDA receptors, AMPA receptors, KA receptors, metabotropic glutamate receptors (mGluRs), GABA(A) receptors, GABA(C) receptors, and glycine receptors during recording. 50 μM L-AP4 will completely activate mGluRs to block the further response induced by presynaptic glutamate release in the retina [2, 22, 31]. Both spontaneous activity and current injection-induced responses were recorded before and during the application of 1 μM oxytocin. Data were obtained using an Axon 700B amplifier, digitized at 10 kHz, filtered at 4 kHz, and collected using the pCLAMP software (Molecular Devices).

### Intravitreal injection

Animals were dark-adapted for 24 h and then deeply anesthetized with 0.6% pentobarbital sodium (15 μl/g). One microliter oxytocin (1 mM, 500 μM, 100 μM, 10 μM, and 1 μM) or 1 μl WAY267464 (1 mM), dissolved with saline solution, was injected into the vitreous of one eye by a NanojectIII microinjector (Drummond Scientific Company, USA) at a speed of ~ 5 nl/s, and the other eye was injected with the same volume of saline as control. The 1 μl volume was chosen based on a previous study [2]. Since 1 mM oxytocin application had a reliable and large effect on the reduction of ERG b-wave, 1 mM oxytocin was used for the experiments to investigate the possible mechanisms. To further verify the results of exogenous oxytocin application, the specific and selective OxtR agonist WAY267464 [25, 80] was used, which has been widely used as the non-peptide agonist to investigate the function of oxytocin signal.

### Measuring retinal dopamine level with high-performance liquid chromatography (HPLC)

About 1 h after oxytocin and saline injection, the retinas or vitreous bodies (collected by an Eppendorf pipette with a 10 μl pipette tip) were harvested [42]. As described previously [81], each frozen sample was homogenized into 100 μl of ice-cold 0.1 M perchloric acid containing 10 μM ascorbic acid, 0.1 mM EDTA disodium salt, and 0.02 μM 3,4-dihydroxybenzyl-amine. Dopamine, DOPAC (3,4-dihydroxyphenylacetic acid), and HVA (Homovanillic acid) levels were measured with the Agilent 1200 series neurotransmitter analyzer (Agilent Technologies, Santa Clara, CA, USA). Data were collected and analyzed by ChemStation (Agilent Technologies).

### cFos immunostaining and electroretinographic (ERG) recording

For the cFos immunostaining experiment, mice were exposed to the environment with light intensity at ~ 1.0 cd/m^2^ for 1 h, and mice were anesthetized during the whole procedure. Then, mice were sacrificed and the retinas were dissected, fixed, and sliced for cFos immunostaining as described previously. Rabbit anti-cFos (1:1000, 5348, Cell Signaling) and mouse anti-TH (1:1000, 22,941, Immunostar) were used to detect cFos expression in retinal DA neurons.

To assess retinal function, ~ 30 min after the intravitreal injection, ERG was measured as previously reported [81]. The whole procedure was conducted in darkness. Mice were kept anesthetized with 0.6% pentobarbital sodium, and the pupils were dilated by compound tropicamide eye drops (Mydrin-p, Santen Pharmaceutical, Japan). Gold wire ring electrodes (3104RC, Roland, Germany) were placed onto the surface of both corneas, and ERGs were acquired by a pre-amplifier (FZG-81, Jia Long Educational Instruments, China) and band-pass filtered (0.1–100 Hz); 3-ms white light flashes were generated by a LED light source (CQ-LU9079, Qianhan Lighting, China) and presented by a custom-built Ganzfeld dome with 5 different stimulus strengths (0.0016 cd·s/m^2^, 0.0388 cd·s/m^2^, 0.31 cd·s/m^2^, 1.47 cd·s/m^2^, and 2.65 cd·s/m^2^). Light stimulation was controlled by a multi-data acquisition card (PCIe 6321, National Instruments, USA) with a LabVIEW-based code. The animals were placed on a thermostatic plate to maintain body temperature during the recording. The amplitudes of ERG a-wave and b-wave were analyzed after recording. OxtR antagonist—L368, 899 (5 mg/kg); vasopressin 1a receptor antagonist—SR49059 (10 mg/kg); dopamine D1 receptor antagonist—SCH23390 (5 mg/kg); or dopamine D2 receptor antagonist—L741, 626 (3 mg/kg)—were given intraperitoneally ~ 30 min prior to the intravitreal injection of oxytocin. The dosage for L368, 899; SR49059; SCH23390; and L741, 626 was determined from previous studies in mice and rats [82–85].

### Quantification and statistical analysis

All image analyses were carried out in ImageJ (FIJI, NIH). The number of neurons and the number of animals used in every experiment are provided in the figure legends. Group data are expressed as the mean ± SEM. Statistical analysis was performed in GraphPad Prism (GraphPad). Normality was evaluated by the Kolmogorov–Smirnov normality test using GraphPad Prism. For two-group comparisons, statistical significance was determined by two-tailed paired or unpaired Student’s *t*-tests, and Wilcoxon signed-rank test or Mann–Whitney test when assumptions for parametric testing were not satisfied. For multiple group comparisons, two-way and one-way analyses of variance (ANOVA) tests were used for normally distributed data, followed by post hoc analyses. For data that were not normally distributed, non-parametric tests for the appropriate group types were used instead. *p* < 0.05 was considered statistically significant.

## Supplementary Information

**Additional file 1: Fig. S1.** The expression of OxtRs in mouse retina. **Fig. S2.** Example images showing OxtR-eYFP neurons are Brn3a (A) or melanopsin (B) positive in the GCL. **Fig. S3.** eYFP^+^ neurons in Oxtr-Cre; Ai3 mice are mainly GABAergic, but not glycinergic amacrine cells. **Fig. S4.** Distribution of OxtR-eYFP neurons in Oxtr-Cre; Ai3 mouse retina. **Fig. S5.** ChAT and CRH ACs are partially co-localized with OxtR-eYFP neurons, and oxytocin elevates DA level. **Fig. S6.** The effects of oxytocin and OxtR antagonist on the amplitude of ERG b-wave. **Fig. S7.** Effects of oxytocin on b-wave amplitude in the presence of V1AR or DA receptor antagonists.

**Additional file 2.** The images of the original, uncropped blots for the OxtR and GAPDH.

## Data Availability

All data generated or analyzed during this study are included in this published article and its supplementary information files.

## Abbreviations

a.u.: Arbitrary units
AC: Amacrine cell
ChAT: Cholinergic
CRH: Corticotrophin-releasing hormone
DA: Dopamine
DOPAC: 3,4-Dihydroxyphenylacetic acid
EDTA: Ethylenediaminetetraacetic acid
ERG: Electroretinographic
eYFP: Enhanced yellow fluorescent protein
FISH: Fluorescent in situ hybridization
GABA: Gamma-aminobutyric acid
GCL: Ganglion cell layer
GFP: Green fluorescent protein
HC: Horizontal cell
HVA: Homovanillic acid
i.p.: Intraperitoneal
IF: Immunofluorescence
INL: Inner nuclear layer
IPL: Inner plexiform layer
ns: Not significant
ONL: Outer nuclear layer
OPL: Outer plexiform layer
Oxt: Oxytocin
OxtR: Oxytocin receptor
PBS: Phosphate-buffered saline
PFA: Paraformaldehyde
PVN: Paraventricular nucleus of the hypothalamus
RGC: Retinal ganglion cell
RPE: Retinal pigment epithelium
SON: Supraoptic nucleus
WB: Western blot
SAL: Saline
TBS: Tris-buffered saline
TH: Tyrosine hydroxylase
VIP: Vasoactive intestinal peptide
