# Supplementary material for: Activation of oxytocin receptors in mouse GABAergic amacrine cells modulates retinal dopaminergic signaling
Source: BMC Biol. 2022 Sep 21;20:205. doi: 10.1186/s12915-022-01405-0 (PMC9490981; doi:10.1186/s12915-022-01405-0)
Supplement: Supplementary file 1 — Additional file 1: Fig. S1. The expression of OxtRs in mouse retina. Fig. S2. Example images showing OxtR-eYFP neurons are Brn3a (A) or melanopsin (B) positive in the GCL. Fig. S3. eYFP+ neurons in Oxtr-Cre; Ai3 mice are mainly GABAergic, but not glycinergic amacrine cells. Fig. S4. Distribution of OxtR-eYFP neurons in Oxtr-Cre; Ai3 mouse retina. Fig. S5. ChAT and CRH ACs are partially co-localized with OxtR-eYFP neurons, and oxytocin elevates DA level. Fig. S6. The effects of oxytocin and OxtR antagonist on the amplitude of ERG b-wave. Fig. S7. Effects of oxytocin on b-wave amplitude in the presence of V1AR or DA receptor antagonists. [file 12915_2022_1405_MOESM1_ESM.docx]

**Supplementary Information**

**Activation of oxytocin receptors in mouse GABAergic amacrine cells modulates retinal dopaminergic signaling**

Songhui Hu^1^*, Yurong Wang^1^*, Xu Han^1^, Min Dai^2^, Yongxing Zhang^1^, Yuanyuan Ma^1^, Shijun Weng^1^, Lei Xiao^1^

^1^The State Key Laboratory of Medical Neurobiology, MOE Frontiers Center for Brain Science, and the Institutes of Brain Science, Fudan University, Shanghai, 200032, China

^2^School of Biomedical Engineering, Shanghai Jiao Tong University, Shanghai 200240, China

* S.H. and Y.W. contributed equally to this work.

Correspondence: leixiao@fudan.edu.cn


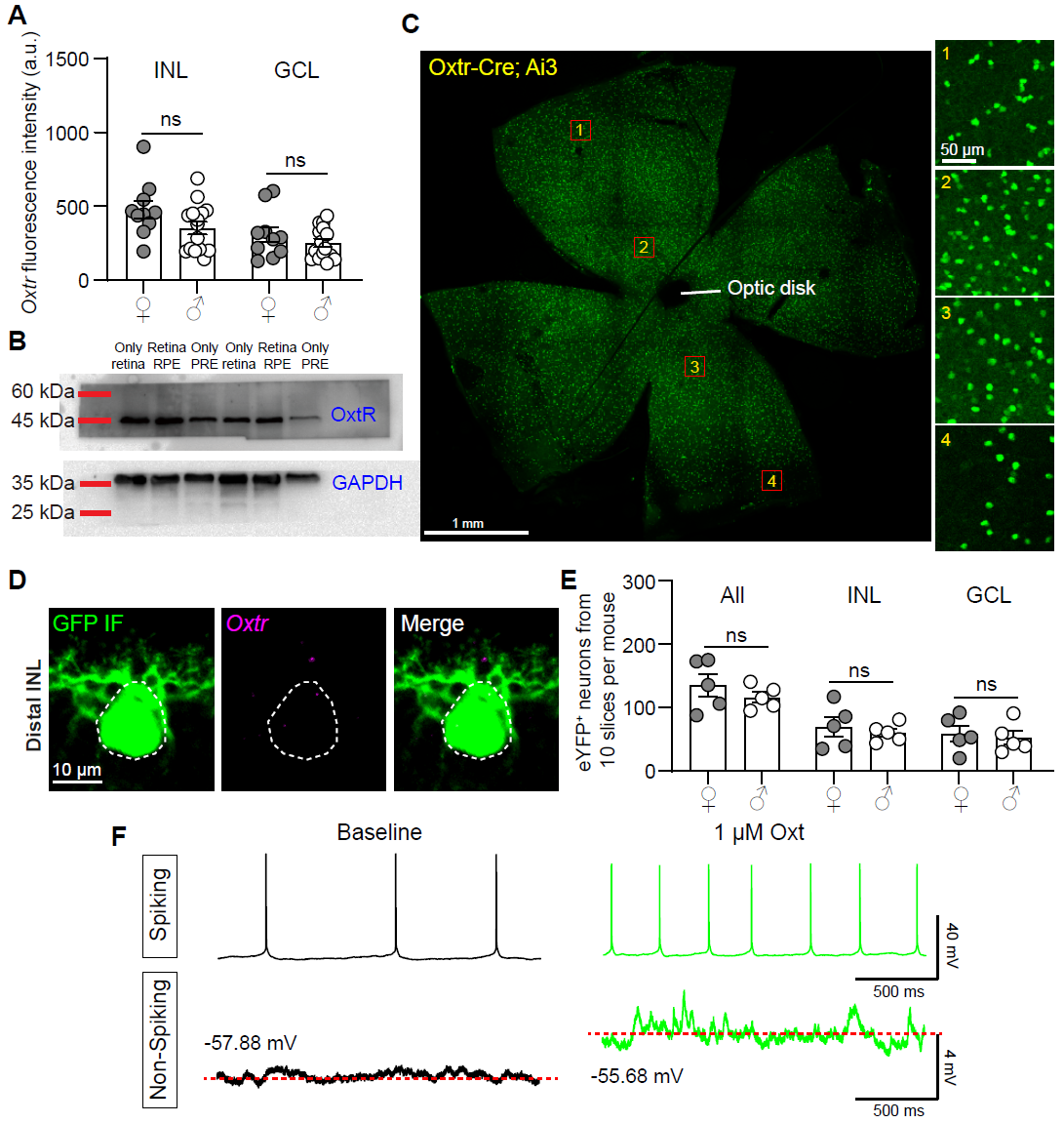


**Figure S1. The expression of OxtRs in mouse retina.** (**A**) Summary of *Oxtr* mRNA fluorescence intensity in the INL and GCL of female (♀) and male (♂) mice. ns indicates not significant, unpaired *t*-test, *n* = 10 slices and 15 slices from 2 female mice and 3 male mice, respectively. (**B**) Western blot imaging shows OxtR protein (Top) and GAPDH protein (Bottom) in the only retina, both retina and RPE, and only RPE from two mice. (**C**) Left: An example image of whole-mount retina from one Oxtr-Cre; Ai3 mouse. Right panels: Magnification images of regions labeled by the red rectangles in Left. (**D**) A confocal image showing few *Oxtr* mRNAs expressed in the eYFP^+^ neuron of distal INL. (**E**) The numbers of eYFP^+^ neurons from Oxtr-Cre; Ai3 female and male mice in the whole retina (All), INL, and GCL. eYFP^+^ neurons were counted from 10 slices per mouse. Unpaired *t*-test, *n* = 5 female mice and 5 male mice. (**F**) Spontaneous response traces of one Spiking eYFP^+^ neuron (Top) and one non-Spiking eYFP^+^ neuron (Bottom) in the GCL before (Left) and during (Right) application of 1 μM oxytocin. Red dashed lines indicate the resting membrane potential levels.


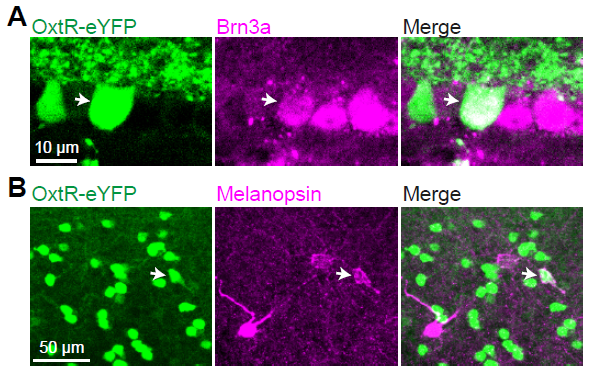


**Figure S2. Example images showing OxtR-eYFP neurons are Brn3a (A) or melanopsin (B) positive in the GCL.**


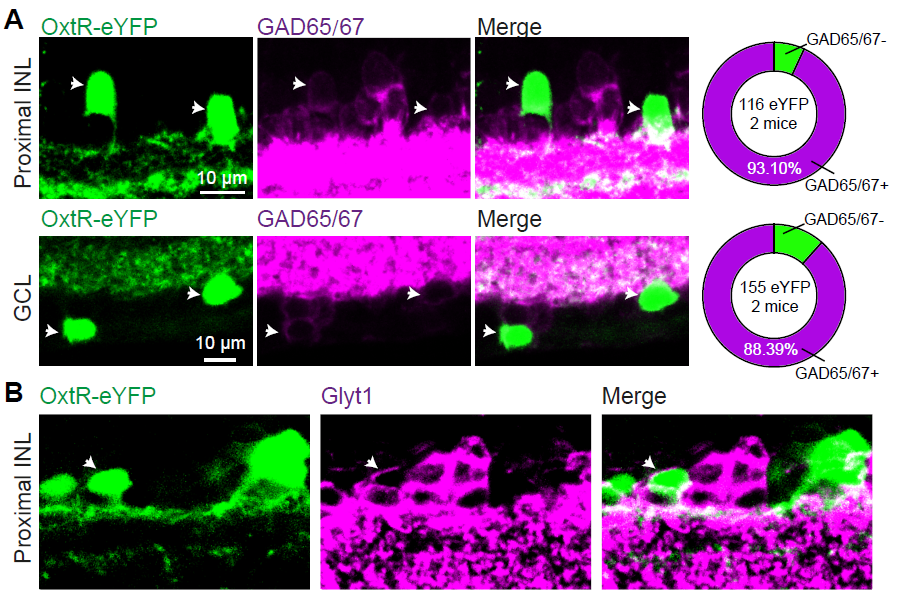


**Figure S3. eYFP^+^ neurons in Oxtr-Cre: Ai3 mice are mainly GABAergic, but not glycinergic amacrine cells.** (**A**) Retinal sections from Oxtr-Cre: Ai3 mice were immunostained for GAD65/67 in INL (**A**, top) and GCL (**A**, bottom). (**B**) An example image showing one eYFP^+^ neuron in Oxtr-Cre: Ai3 mouse is Glyt1 positive.


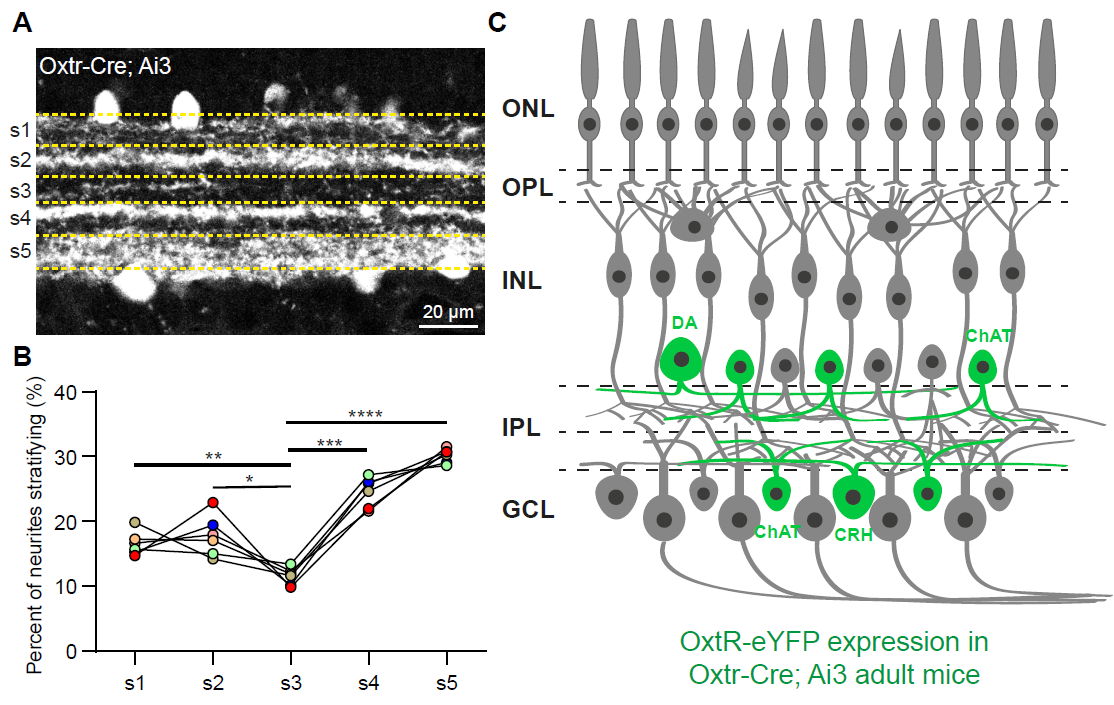


**Figure S4. Distribution of OxtR-eYFP neurons in Oxtr-Cre; Ai3 mouse retina.** (**A**) An example image of retinal IPL from one Oxtr-Cre; Ai3 mouse showing the location of eYFP^+^ AC dendrites in the five strata (s1-s5) of retinal IPL. (**B**) Summary about the percentage of neurite stratifications in retinal IPL. The percentage of neurite stratifications is defined as the ratio between average fluorescence intensity in each stratum and the sum of the fluorescence intensity in the IPL. *n* = 7 mice. **p* < 0.05, ***p* < 0.01, ****p* < 0.001, *****p* < 0.0001, one way ANOVA with Holm-Sidak *post hoc* tests. (**C**) Schematic image showing the expression of OxtRs in the adult mouse retina. Our study found that OxtRs are mainly expressed in DA, ChAT, and CRH ACs in INL and GCL in adult mice.


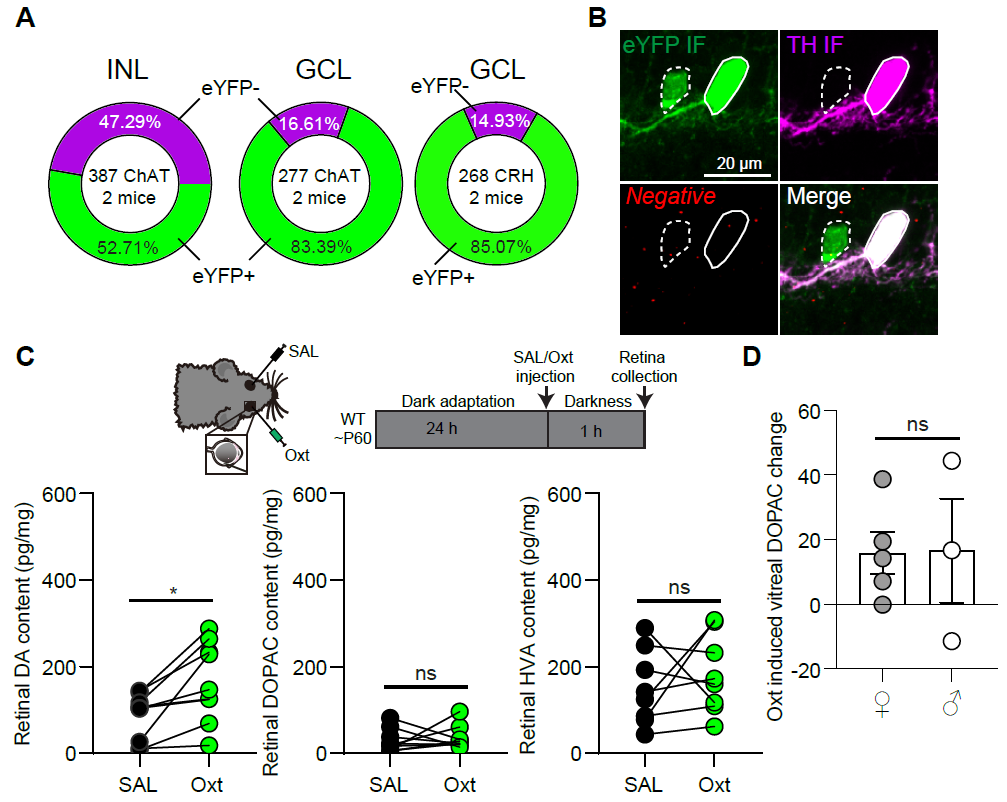


**Figure S5. ChAT and CRH ACs are partially co-localized with OxtR-eYFP neurons, and oxytocin elevates DA level.** (**A**) Summary results showing the ratio of INL ChAT (Left), GCL ChAT (Middle), and GCL CRH (Right) neurons co-localized with eYFP^+^ neurons in Oxtr-Cre; Ai3 mice. (**B**) Confocal images showing co-localization of eYFP^+^ neuron (GFP IF), TH^+^ neuron, and *negative* control probe in FISH. White solid and dashed circles indicate TH^+^ and TH^−^ neurons, respectively. (**C**) Summaries of retinal DA (Left), DOPAC (Middle), and HVA (Right) with intravitreal injecting SAL and Oxt under darkness environment. **p* < 0.05, Wilcoxon matched-pairs signed rank test, *n* = 9 mice. (**D**) Summary of the vitreal DOPAC level change induced by oxytocin in female and male mice. *p* > 0.05, Mann-Whitney test, *n* = 5 females and 3 males.


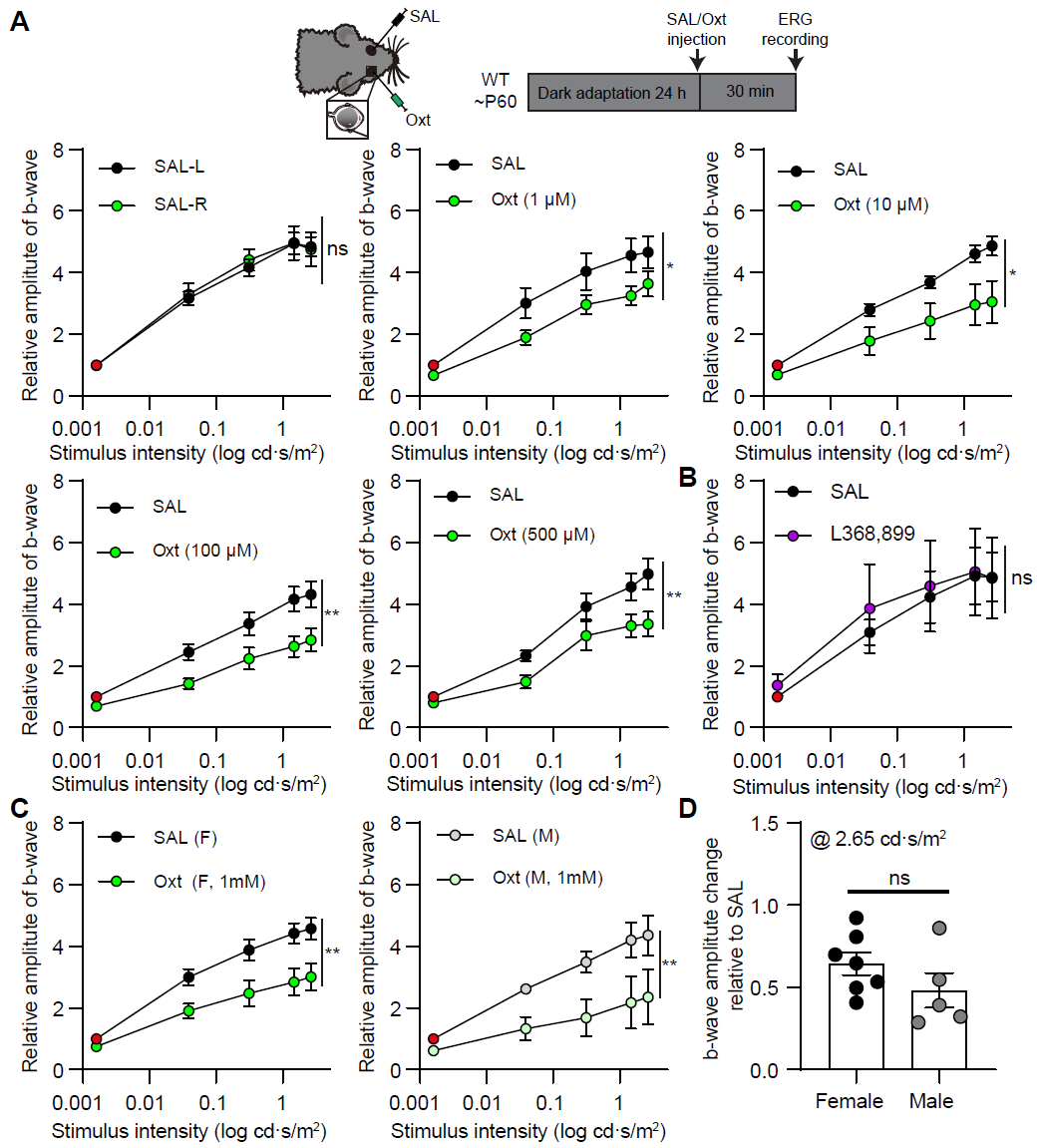


**Figure S6. The effects of oxytocin and OxtR antagonist on the amplitude of ERG b-wave.** (**A**) Top: Schematic of intravitreal injection and ERG recording protocol. Middle and bottom: Effects of saline, 1 μM Oxt, 10 μM Oxt, 100 μM Oxt, and 500 μM Oxt on the relative amplitude of ERG b-wave. ERG b-wave amplitudes were normalized to the amplitude of b-wave recorded from the eye with SAL injection and the stimulation intensity at 0.0016 cd·s/m^2^, labeled with red circles. *n* = 6, 9, 7, 8, and 7 mice for saline, 1 μM, 10 μM, 100 μM, and 500 μM, respectively. * *p* < 0.05, ** *p* < 0.01, two-way ANOVA test. (**B**) Effect of 500 μM oxytocin receptor antagonist L368, 899 intravitreal injection on the relative amplitude of ERG b-wave. *n* = 6 mice, *p* > 0.05, two-way ANOVA test. (**C**) Effect of 1 mM Oxt on the relative amplitude of ERG b-wave in females (Left) and males (Right). *n* = 7 female and 5 male mice, ** *p* < 0.01, two-way ANOVA test. (**D**) 1 mM oxytocin-induced b-wave amplitude change in the females and males. *n* = 7 female and 5 male mice, *p* = 0.2331, unpaired *t*-test.


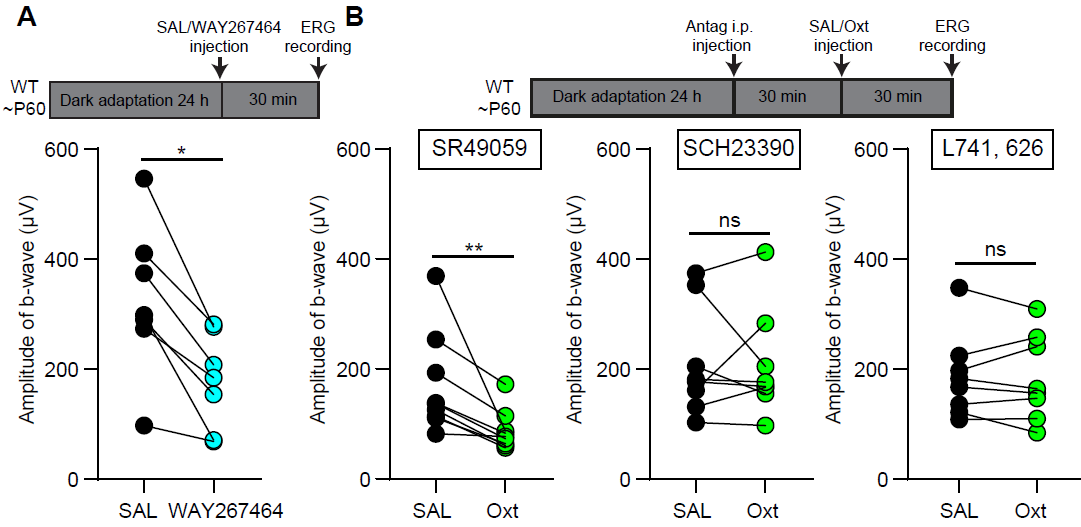


**Figure S7. Effects of oxytocin on b-wave amplitude in the presence of V1AR or DA receptor antagonists.** (**A**) WAY267464 application significantly reduced the amplitude of ERG b-wave with light stimulation intensity at 2.65 cd·s/m^2^. *n* = 7 mice, **p* < 0.01, paired *t*-test. (**B**) For the mice pretreated with V1AR blocker – SR49059 (*n* = 9 mice), D1 receptor antagonist – SCH23390 (*n* = 8 mice), or D2 receptor antagonist – L741, 626 (*n* = 8 mice), the effect of oxytocin application on the amplitude of ERG b-wave with light stimulation intensity at 2.65 cd·s/m^2^. ** *p* < 0.01 for SR49059, Wilcoxon matched-pairs signed-rank test; *p* = 0.9320 and 0.5770 for SCH23390 and L741, 626, respectively, paired *t*-test.
