# Supplementary figures and images for: Activation of oxytocin receptors in mouse GABAergic amacrine cells modulates retinal dopaminergic signaling
Source: BMC Biol. 2022 Sep 21;20:205. doi: 10.1186/s12915-022-01405-0 (PMC9490981; doi:10.1186/s12915-022-01405-0)

OxtR

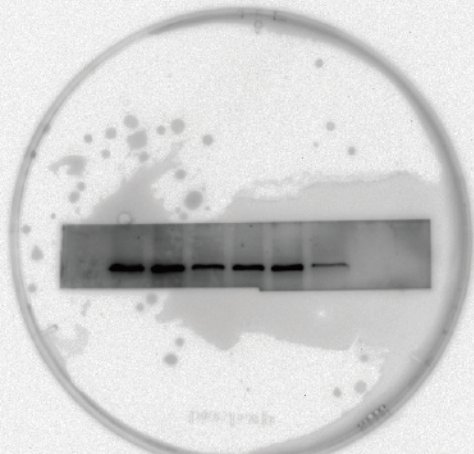

GAPDH

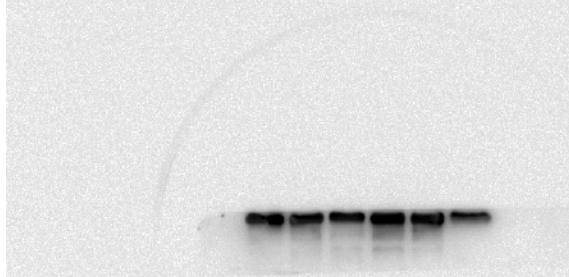

Supplement: Supplementary file 2 — Additional file 2. The images of the original, uncropped blots for the OxtR and GAPDH. [file 12915_2022_1405_MOESM2_ESM.pdf]
